# Guest Editorial

**Published:** 2015-04-28

**Authors:** Neeraj Gugnani

**Affiliations:** MDS, MSc (Clinical Trials, LSHTM, UoL), Professor, Department of Pedodontics and PCD, DAV(C) Dental College, Yamuna Nagar, Haryana, India

## Abstract

Despite the advancements in dentistry, dental caries still remains the most common disease of the oral cavity. A major reason for this scenario is because the dental professionals are still clinging to the outdated surgical model of dental caries. This traditional approach of caries management forces the tooth to enter into the ‘restoration cycle’ which usually involves several lifetime replacement procedures, resulting in increased restoration size, even more invasive procedures and finally a prosthesis. We need to acknowledge that, by simply drilling and filling the carious lesions, this disease cannot be controlled. Like other infectious diseases of the human body, if the etiological factors are not identified and managed appropriately, the disease will continue.

Early Caries Detection and Caries Risk Assessment… A must-do for All Patients!!

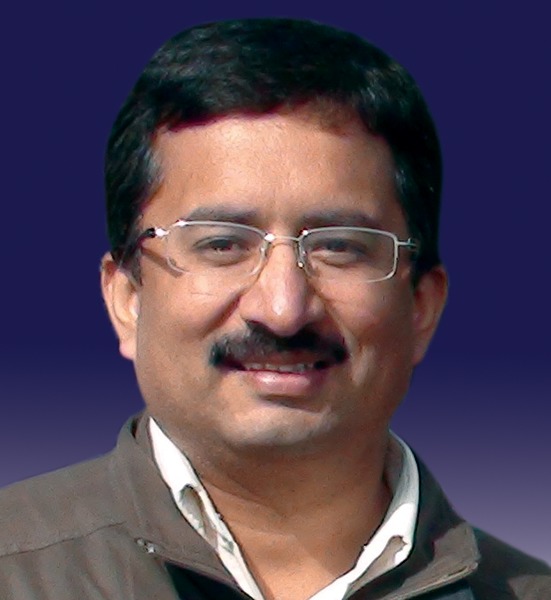



Despite the advancements in dentistry, dental caries still remains the most common disease of the oral cavity. A major reason for this scenario is because the dental professionals are still clinging to the outdated surgical model of dental caries. This traditional approach of caries management forces the tooth to enter into the ‘restoration cycle’ which usually involves several lifetime replacement procedures, resulting in increased restoration size, even more invasive procedures and finally a prosthesis. We need to acknowledge that, by simply drilling and filling the carious lesions, this disease cannot be controlled. Like other infectious diseases of the human body, if the etiological factors are not identified and managed appropriately, the disease will continue.

Dental caries needs to be viewed as a continuum of disease caused by specific pathogens, with patients at varying degrees of risk. We should always remember that caries is the consequence of a shift in the homeostatic balance of the resident microfora due to a change in the local environmental conditions that favor the cariogenic pathogens growth. Just by repairing the carious lesion(s), dental caries disease is not fully treated, because the actual causes and the risk factors are not fully resolved.

Detecting ‘noncavitated lesions’ along with the cavitated ones and assessing the caries risk are two critical components of dental caries management, and should be considered a standard of care for all patients. These should always be included as a part of routine dental examination and customized individual treatment plan should be made for all patients, taking patient’s caries risk into consideration. Risk categorization helps the clinician in the decisionmaking process concerning preventive strategies, treatment, recall appointments. Moreover, treatments based on risk assessment are more successful and cost-effective compared with the traditional approach.

Hence, clinicians, dental students and researchers should divert their energies in learning and implementing the two basic rules: detect early caries and treat after assessing the caries risk. We have to bid adieu to our old traditional approach and move to the new horizons.

                                                                                                                                    **Best Regards**

                                                                                                                                    Neeraj Gugnani

                                                                                             MDS, MSc (Clinical Trials, LSHTM, UoL)

                                                                                   Professor, Department of Pedodontics and PCD

                                                                                                                       DAV(C) Dental College

                                                                                                            Yamuna Nagar, Haryana, India

